# Nirmatrelvir methyl *tert*-butyl ether solvate

**DOI:** 10.1107/S2056989026003816

**Published:** 2026-04-14

**Authors:** Jared P. Smit, Dale K. Purcell, David A. Engers, Pamela A. Smith, Haley C. Bauser, Adrian Radocea

**Affiliations:** a Improved Pharma, LLC, 1281 Win Hentschel Blvd., West Lafayette, IN 47906, USA; b Varda Space Industries, 225 S. Aviation Blvd., El Segundo, CA 90245, USA; Texas A & M University, USA

**Keywords:** crystal structure, pharmaceutical, nirmatrelvir

## Abstract

The structure of nirmatrelvir MTBE solvate, C_23_H_32_F_3_N_5_O_4_·C_5_H_12_O, at 150 K has monoclinic (*P*2_1_) symmetry. The asymmetric unit contains one nirmatrelvir mol­ecule and one methyl *tert*-butyl ether solvent mol­ecule. The extended structure consists of N—H⋯O hydrogen bonds that create create [010] chains. Crystals of the title compound were grown from various methyl *tert*-butyl ether–methanol solvent mixtures, with crystal morphology dependent upon the mixture ratio.

## Abstract 2

The structure of nirmatrelvir MTBE solvate has monoclinic (*P*2_1_) symmetry. The asymmetric unit contains one nirmatrelvir mol­ecule and one methyl *tert*-butyl ether mol­ecule. The extended structure consists of N—H⋯O hydrogen bonds that create [010] chains.

## 1. Chemical context

Nirmatrelvir (NTV) is an anti­viral protease inhibitor developed by Pfizer, currently used in tandem with ritonavir and marketed as Paxlovid^®^ for the treatment of Covid-19 (Halford, 2022 ▸; Lamb, 2022 ▸). Two enanti­otropically related anhydrous polymorphs (designated Forms 1 and 4) of NTV have been well characterized, and the higher temperature stable form, Form 1, was selected for use in the drug product (Sadeghi *et al.*, 2024 ▸). A crystalline X-ray powder diffraction (XRPD) pattern for Form 2, a methyl *tert*-butyl ether (MTBE) solvate, was published in a US patent although the Form 2 crystal structure was not disclosed (Owens *et al.*, 2022 ▸). Form 2 is of inter­est as a key inter­mediate isolated in the purification process, enabling the subsequent generation of the stable, non-solvated Form 1 through recrystallization. The crystal structure of NTV Form 2, a mono-MTBE solvate, is reported in this work.
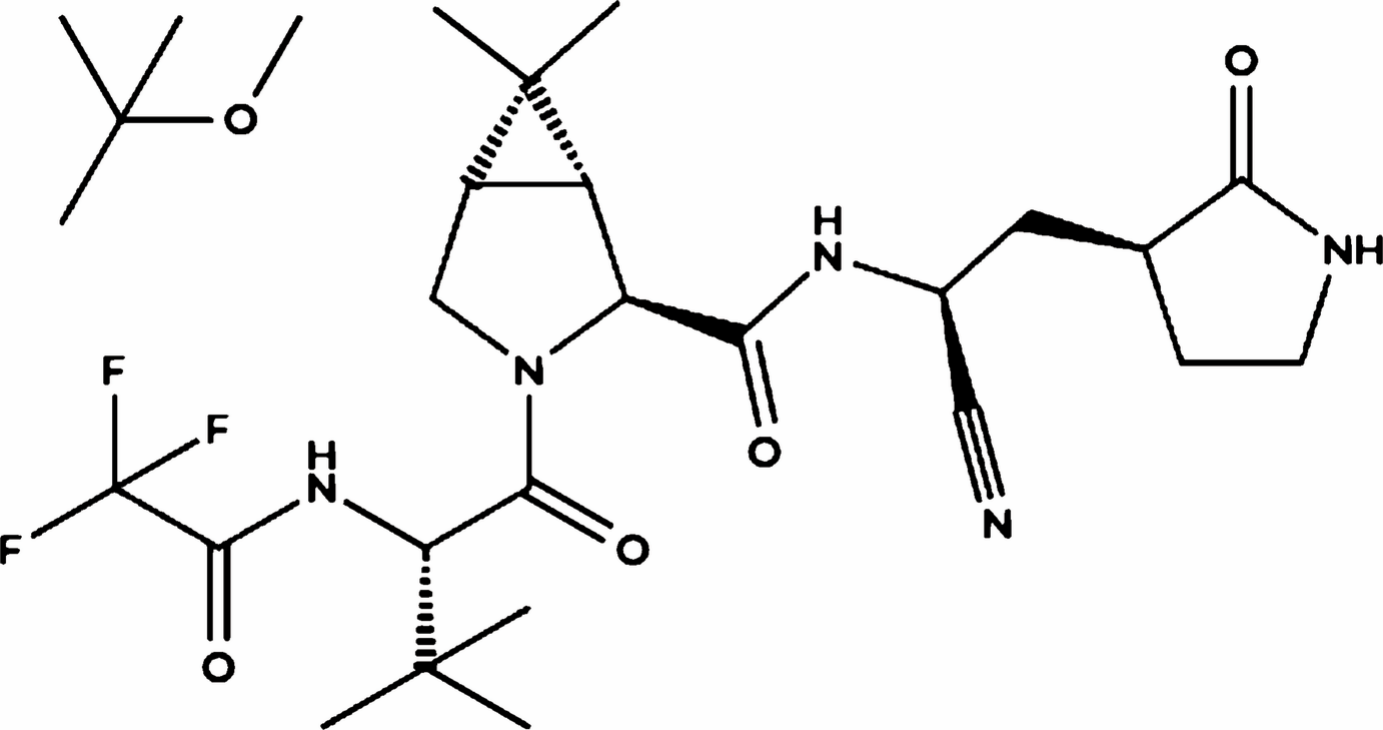


## 2. Structural commentary

**Figure 1:**
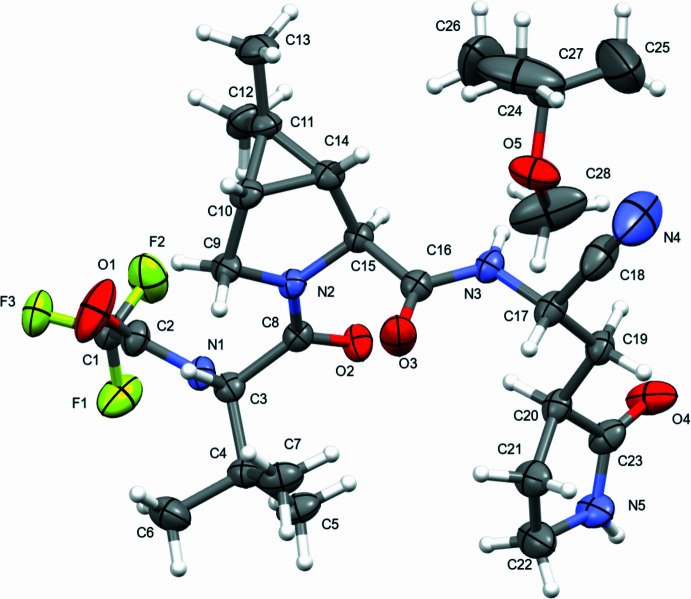
The title mol­ecule with displacement ellipsoids drawn at the 50% probability level. Solvent disorder is not shown for clarity.

The mol­ecular structure of NTV is shown in Fig. 1 ▸. Nirmatrelvir Form 2 is a mono-MTBE solvate, crystallizing in space group *P*2_1_, with an asymmetric unit consisting of one NTV mol­ecule and one MTBE solvent mol­ecule. The MTBE solvent mol­ecule is disordered over two conformations that are approximate mirror images of each other, refining to 69% in the predominant orientation. The absolute structure was determined from the data and the NTV molecule was found to bond in the *S,S*, *R*, *S*, *S*, and *S* configuration at C3, C10, C14, C15, C17, and C20 respectively.

## 3. Supra­molecular features

**Figure 2:**
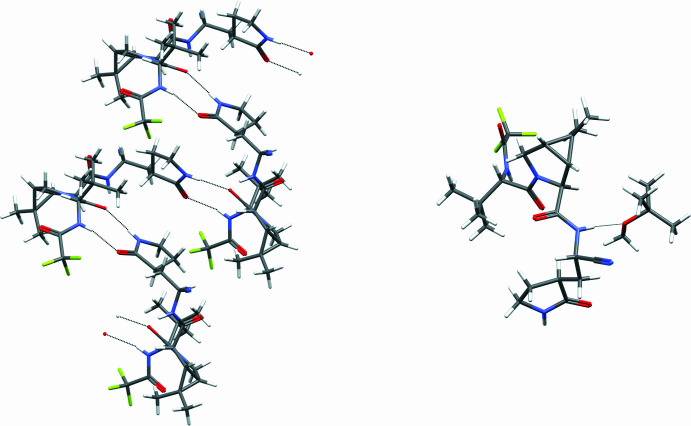
One-dimensional hydrogen-bonded chain of nirmatrelvir mol­ecules (left) and NTV hydrogen bonded to MTBE (right).

Nirmatrelvir contains three secondary amide moieties that are available for hydrogen-bond donation. The pyrrolidone amide forms a hydrogen bond to the pyrrolidine amide carbonyl and accepts a hydrogen bond from the tri­fluoro­acetyl amide from an adjacent mol­ecule, creating hydrogen bonded chains propagating along the *b-*axis direction, shown in Fig. 2 ▸. The pyrimidine NH grouping forms a hydrogen bond to the oxygen atom of the MTBE, but this supra­molecular feature does not contribute to the dimensionality. Geometric details of hydrogen bonds are given in Table 1 ▸.

## 4. Database survey

Two anhydrous polymorphs are in the Cambridge Structural Database (CSD, version 6.01, update 1, February 2026; Groom *et al.*, 2016 ▸), refcodes ZIVMEA through ZIVMEA10 (Sadeghi *et al.*, 2024 ▸; Jiang *et al.*, 2023 ▸).. The two forms are enanti­otropic polymorphs, both crystallizing in space group *P*2_1_2_1_2_1_, and the mol­ecular packing is similar enough that the two forms can undergo a first order solid-state single crystal-to-single crystal phase transition (Shi *et al.*, 2025 ▸). The pyrrolidone amide forms and accepts hydrogen bonds to and from the same amide oxygen moiety on two different adjacent mol­ecules rather than the hydrogen bonding to the pyrrolidine amide and from the tri­fluoro­acetyl amide on one adjacent mol­ecule in the framework of the title solvate.

Isostructural ethanol and iso­propanol solvates (refcodes AMEPOB and AMESOE) have also been reported (Li *et al.*, 2026 ▸). These solvates have three NTV mol­ecules in the asymmetric unit, each forming different hydrogen bonds. The pyrrolidone amide moiety on one of the three unique mol­ecules forms the same hydrogen bonds as in the structure of the MTBE solvate.

## 5. Synthesis and crystallization

**Figure 3:**
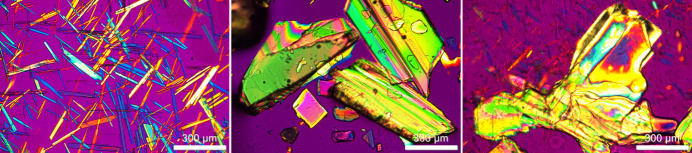
Morphology of NTV MTBE solvate crystals at MTBE:methanol ratios of 99:1, 96:4, and 95:5 (left to right)

Nirmatrelvir was obtained from ChemShuttle and used without purification. Single crystals were obtained from MTBE and from solutions of 94 to 99% (*v*/*v*) MTBE in methanol. The solid (0.02 g) was dissolved in each MTBE solution. After standing at room temperature overnight, a solid precipitate was observed. Solubilities of NTV in each solution were determined gravimetrically from supernatant filtered through a 0.2 mm PTFE membrane into clean pre-tared vials. The measured solubilities ranged from 0.7 mg ml^−1^ in MTBE to 6.5mg ml^−1^ in 94% (*v*/v) MTBE–methanol solution. After standing at room temperature for an extended time (18 months), crystals suitable for single crystal X-ray diffraction (SCXRD) were discovered and isolated. The apparent morphology of the crystals varied with solution composition, with finer blades and needle-shaped crystals obtained at 99% (*v*/*v*) MTBE and larger block-shaped and prismatic crystals obtained as methanol concentration increased, 95–96% (*v*/*v*) MTBE, shown in polarized light microscopy images (Fig. 3 ▸).

Polarized light microscopy was conducted using an Olympus Series BX51TRF (Olympus America Inc., Melville, NY) equipped with 12 V/100 W transmission and reflection illumination, an achromat 0.9 NA polarized light condenser, Olympus Series MPlanFL N objectives: 50X/0.75 NA BDP, 20X/0.40 NA BDP, 10X/0.25 NA BDP, 5X/0.15 NA BDP, an inter­mediate tube with variable position analyzer and compensator, a trinocular viewing head with a Lumenera Series Infinity 3-3URC (Teledyne Lumenera, Ottawa, Ontario, Canada) digital camera. Image capture and image processing using Image-Pro® version 10.0.11 Build 7240 (Date: 01-April-2020).

A small portion of the sample was pipetted into the well of a scrupulously cleaned depression microscope slide and a No. 1 ½ cover glass was placed over the sample, which was sealed using grease to prevent fast of the volatile solvent.

The relatively larger amount of the more polar methanol in MTBE increased the solubility and therefore decreased the nucleation rate, leading to fewer, but larger, crystals. Face indexing using *CrysAlis PRO* (Rigaku OD, 2026 ▸) indicates that the needles are growing faster along the [010] direction, consistent with the direction of hydrogen bonding and therefore the presence of the hydrogen-bond donating methanol slows that process (McArdle & Erxleben, 2024 ▸). Crystals from each solution were indexed with unit-cell parameters matching the crystal structure of the MTBE solvate. The XRPD pattern calculated from the crystal structure is consistent with the powder pattern previously published (Owens *et al.*, 2022 ▸).

## 6. Refinement

Crystal data, data collection and structure refinement details are summarized in Table 2 ▸. Hydrogen atoms residing on nitrogen were refined independently. Hydrogen atoms residing on carbon were included in the refinement using the appropriate riding models. The MTBE molecule is disordered over two orientations that are related through a pseudo-inversion, refining to 69% in the predominant orientation, and modeled using restraints to the anisotropic displacement parameters (SIMU/DELU).

## Supplementary Material

Crystal structure: contains datablock(s) I. DOI: 10.1107/S2056989026003816/jy2071sup1.cif

Structure factors: contains datablock(s) I. DOI: 10.1107/S2056989026003816/jy2071Isup2.hkl

Supporting information file. DOI: 10.1107/S2056989026003816/jy2071Isup3.mol

CCDC reference: 2545296

Additional supporting information:  crystallographic information; 3D view; checkCIF report

## Figures and Tables

**Table 1:** Hydrogen-bond geometry (Å, °)

*D*—H⋯*A*	*D*—H	H⋯*A*	*D*⋯*A*	*D*—H⋯*A*
N1—H1⋯O4^i^	0.80 (3)	2.06 (3)	2.837 (2)	165 (4)
N3—H3⋯O5	0.87 (3)	1.99 (3)	2.848 (4)	170 (2)
N3—H3⋯O5*A*	0.87 (3)	1.95 (3)	2.795 (10)	163 (3)
N5—H5⋯O2^ii^	0.85 (3)	2.00 (3)	2.845 (2)	176 (3)

Symmetry codes: (i) 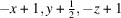; (ii) 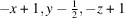.

**Table 2:** Experimental details

Crystal data	
Chemical formula	C_23_H_32_F_3_N_5_O_4_·C_5_H_12_O
*M*_r_	587.68
Crystal system, space group	Monoclinic, *P*2_1_
Temperature (K)	150
*a*, *b*, *c* (Å)	12.7655 (1), 9.2588 (1), 14.3951 (1)
β (°)	103.208 (1)
*V* (Å^3^)	1656.40 (3)
*Z*	2
Radiation type	Cu *K*α
μ (mm^−1^)	0.78
Crystal size (mm)	0.35 × 0.24 × 0.12

Data collection	
Diffractometer	XtaLAB Synergy, Single source at home/near, HyPix-Bantam
Absorption correction	Multi-scan (*CrysAlis PRO*; Rigaku OD, 2026 ▸)
*T*_min_, *T*_max_	0.691, 1.000
No. of measured, independent and observed [*I* > 2σ(*I*)] reflections	31765, 5959, 5890
*R*_int_	0.023
(sin θ/λ)_max_ (Å^−1^)	0.604

Refinement	
*R*[*F*^2^ > 2σ(*F*^2^)], *wR*(*F*^2^), *S*	0.035, 0.094, 1.03
No. of reflections	5959
No. of parameters	442
No. of restraints	46
H-atom treatment	H atoms treated by a mixture of independent and constrained refinement
Δρ_max_, Δρ_min_ (e Å^−3^)	0.29, −0.20
Absolute structure	Flack *x* determined using 2630 quotients [(*I*^+^)−(*I*^−^)]/[(*I*^+^)+(*I*^−^)] (Parsons *et al.*, 2013 ▸)
Absolute structure parameter	−0.03 (3)

Computer programs: *CrysAlis PRO* (Rigaku OD, 2026 ▸), *SHELXT* (Sheldrick, 2015*a* ▸), *SHELXL2018/3* (Sheldrick, 2015*b* ▸) and *OLEX2* (Dolomanov *et al.*, 2009 ▸).

## supplementary crystallographic information

### (1R,2S,5S)-N-[(1S)-1-Cyano-2-[(3S)-2-oxopyrrolidin-3-yl]ethyl]-3-[(2S)-3,3-dimethyl-2-[(2,2-trifluoroacetyl)amino]butanoyl]-6,6-dimethyl-3-azabicyclohexane-2-carboxamide methyl <it>tert</it>-butyl ether monosolvate Crystal data

**Table:**

C_23_H_32_F_3_N_5_O_4_·C_5_H_12_O	*F*(000) = 628
*M**_r_* = 587.68	*D*_x_ = 1.178 Mg m^−^^3^
Monoclinic, *P*2_1_	Cu *K*α radiation, λ = 1.54184 Å
*a* = 12.7655 (1) Å	Cell parameters from 28535 reflections
*b* = 9.2588 (1) Å	θ = 3.2–68.6°
*c* = 14.3951 (1) Å	µ = 0.78 mm^−^^1^
β = 103.208 (1)°	*T* = 150 K
*V* = 1656.40 (3) Å^3^	Block, colourless
*Z* = 2	0.35 × 0.24 × 0.12 mm

### (1R,2S,5S)-N-[(1S)-1-Cyano-2-[(3S)-2-oxopyrrolidin-3-yl]ethyl]-3-[(2S)-3,3-dimethyl-2-[(2,2-trifluoroacetyl)amino]butanoyl]-6,6-dimethyl-3-azabicyclohexane-2-carboxamide methyl <it>tert</it>-butyl ether monosolvate Data collection

**Table:**

XtaLAB Synergy, Single source at home/near, HyPix-Bantam diffractometer	5959 independent reflections
Radiation source: micro-focus sealed X-ray tube, PhotonJet (Cu) X-ray Source	5890 reflections with *I* > 2σ(*I*)
Mirror monochromator	*R*_int_ = 0.023
Detector resolution: 10.0000 pixels mm^-1^	θ_max_ = 68.7°, θ_min_ = 3.2°
ω scans	*h* = −15→15
Absorption correction: multi-scan (CrysAlisPro; Rigaku OD, 2026)	*k* = −11→11
*T*_min_ = 0.691, *T*_max_ = 1.000	*l* = −17→17
31765 measured reflections	

### (1R,2S,5S)-N-[(1S)-1-Cyano-2-[(3S)-2-oxopyrrolidin-3-yl]ethyl]-3-[(2S)-3,3-dimethyl-2-[(2,2-trifluoroacetyl)amino]butanoyl]-6,6-dimethyl-3-azabicyclohexane-2-carboxamide methyl <it>tert</it>-butyl ether monosolvate Refinement

**Table:**

Refinement on *F*^2^	Hydrogen site location: mixed
Least-squares matrix: full	H atoms treated by a mixture of independent and constrained refinement
*R*[*F*^2^ > 2σ(*F*^2^)] = 0.035	*w* = 1/[σ^2^(*F*_o_^2^) + (0.062*P*)^2^ + 0.2608*P*] where *P* = (*F*_o_^2^ + 2*F*_c_^2^)/3
*wR*(*F*^2^) = 0.094	(Δ/σ)_max_ = 0.001
*S* = 1.03	Δρ_max_ = 0.29 e Å^−^^3^
5959 reflections	Δρ_min_ = −0.20 e Å^−^^3^
442 parameters	Absolute structure: Flack *x* determined using 2630 quotients [(*I*^+^)-(*I*^-^)]/[(*I*^+^)+(*I*^-^)] (Parsons *et al.*, 2013)
46 restraints	Absolute structure parameter: −0.03 (3)
Primary atom site location: dual	

### (1R,2S,5S)-N-[(1S)-1-Cyano-2-[(3S)-2-oxopyrrolidin-3-yl]ethyl]-3-[(2S)-3,3-dimethyl-2-[(2,2-trifluoroacetyl)amino]butanoyl]-6,6-dimethyl-3-azabicyclohexane-2-carboxamide methyl <it>tert</it>-butyl ether monosolvate Special details

**Table:**

Geometry. All esds (except the esd in the dihedral angle between two l.s. planes) are estimated using the full covariance matrix. The cell esds are taken into account individually in the estimation of esds in distances, angles and torsion angles; correlations between esds in cell parameters are only used when they are defined by crystal symmetry. An approximate (isotropic) treatment of cell esds is used for estimating esds involving l.s. planes.

### (1R,2S,5S)-N-[(1S)-1-Cyano-2-[(3S)-2-oxopyrrolidin-3-yl]ethyl]-3-[(2S)-3,3-dimethyl-2-[(2,2-trifluoroacetyl)amino]butanoyl]-6,6-dimethyl-3-azabicyclohexane-2-carboxamide methyl <it>tert</it>-butyl ether monosolvate Fractional atomic coordinates and isotropic or equivalent isotropic displacement parameters (Å^2^)

**Table:**

	*x*	*y*	*z*	*U*_iso_*/*U*_eq_	Occ. (<1)
F3	0.71689 (12)	0.98226 (17)	0.18387 (10)	0.0502 (4)	
O2	0.42888 (11)	0.49248 (18)	0.29537 (9)	0.0346 (3)	
F2	0.63537 (16)	0.9574 (2)	0.29654 (13)	0.0622 (5)	
F1	0.77491 (12)	0.82994 (19)	0.29599 (12)	0.0575 (4)	
N2	0.34108 (12)	0.54106 (18)	0.14552 (11)	0.0260 (3)	
N1	0.60011 (13)	0.6527 (2)	0.22920 (12)	0.0291 (4)	
O3	0.24679 (16)	0.2686 (2)	0.13660 (13)	0.0480 (4)	
O4	0.32501 (18)	0.1791 (2)	0.57021 (11)	0.0584 (6)	
N3	0.16245 (13)	0.3324 (2)	0.25251 (12)	0.0308 (4)	
N5	0.45298 (16)	0.0532 (3)	0.51601 (13)	0.0431 (5)	
O1	0.5744 (2)	0.7964 (3)	0.09820 (14)	0.0756 (8)	
C1	0.68689 (17)	0.8859 (3)	0.23966 (15)	0.0358 (5)	
C8	0.43156 (15)	0.5203 (2)	0.21144 (13)	0.0264 (4)	
C15	0.23765 (14)	0.5218 (2)	0.17219 (13)	0.0265 (4)	
H15	0.236566	0.576525	0.231853	0.032*	
C16	0.21747 (15)	0.3610 (2)	0.18512 (14)	0.0300 (4)	
C2	0.61382 (17)	0.7711 (3)	0.18146 (14)	0.0341 (4)	
C3	0.53816 (14)	0.5313 (2)	0.18022 (13)	0.0288 (4)	
H3A	0.520209	0.554521	0.110540	0.035*	
C23	0.36464 (19)	0.1326 (3)	0.50598 (15)	0.0377 (5)	
C10	0.21163 (16)	0.6146 (2)	0.00894 (14)	0.0324 (4)	
H10	0.174056	0.589426	−0.057936	0.039*	
C14	0.15510 (15)	0.5825 (2)	0.08827 (14)	0.0316 (4)	
H14	0.082198	0.536855	0.071303	0.038*	
C17	0.14115 (18)	0.1852 (3)	0.27582 (15)	0.0363 (5)	
H17	0.169676	0.119004	0.232567	0.044*	
C11	0.16674 (18)	0.7362 (3)	0.05803 (15)	0.0366 (5)	
C9	0.32856 (16)	0.5740 (3)	0.04271 (13)	0.0311 (4)	
H9A	0.346067	0.488590	0.007773	0.037*	
H9B	0.375806	0.655132	0.033691	0.037*	
C20	0.31739 (16)	0.1561 (3)	0.39984 (13)	0.0333 (4)	
H20	0.338981	0.253910	0.381412	0.040*	
C19	0.19543 (17)	0.1480 (3)	0.37928 (15)	0.0356 (5)	
H19A	0.169260	0.215436	0.422334	0.043*	
H19B	0.174017	0.049149	0.393515	0.043*	
O5	0.1325 (3)	0.5375 (4)	0.3916 (2)	0.0557 (10)	0.687 (8)
C4	0.60242 (17)	0.3874 (3)	0.19259 (15)	0.0355 (5)	
C21	0.3745 (2)	0.0415 (3)	0.35336 (15)	0.0457 (6)	
H21A	0.386753	0.075998	0.291617	0.055*	
H21B	0.332176	−0.049036	0.342658	0.055*	
C12	0.2382 (2)	0.8424 (3)	0.12260 (18)	0.0461 (5)	
H12A	0.307656	0.796793	0.150239	0.069*	
H12B	0.203839	0.871835	0.173883	0.069*	
H12C	0.249686	0.927433	0.085634	0.069*	
C5	0.6507 (2)	0.3496 (3)	0.29742 (17)	0.0448 (6)	
H5A	0.696781	0.429029	0.327825	0.067*	
H5B	0.693608	0.261189	0.300766	0.067*	
H5C	0.592597	0.334485	0.330615	0.067*	
C7	0.5265 (2)	0.2675 (3)	0.14520 (18)	0.0443 (5)	
H7A	0.469853	0.253609	0.180198	0.066*	
H7B	0.567102	0.177540	0.145900	0.066*	
H7C	0.493925	0.294566	0.079088	0.066*	
C22	0.4812 (2)	0.0190 (4)	0.42612 (18)	0.0550 (7)	
H22A	0.506473	−0.081976	0.425368	0.066*	
H22B	0.537516	0.084957	0.413796	0.066*	
C13	0.0639 (2)	0.8043 (4)	0.0006 (2)	0.0557 (7)	
H13A	0.081874	0.878817	−0.041587	0.084*	
H13B	0.024111	0.848199	0.044097	0.084*	
H13C	0.019303	0.729924	−0.037838	0.084*	
C6	0.6935 (2)	0.4043 (3)	0.1404 (2)	0.0514 (6)	
H6A	0.662914	0.420029	0.072286	0.077*	
H6B	0.737522	0.316514	0.148864	0.077*	
H6C	0.738318	0.487166	0.166663	0.077*	
C18	0.0234 (2)	0.1619 (3)	0.2606 (2)	0.0582 (8)	
N4	−0.0664 (2)	0.1441 (4)	0.2507 (3)	0.0961 (13)	
C27	0.0365 (3)	0.6271 (5)	0.3776 (3)	0.0502 (12)	0.687 (8)
C28	0.2129 (10)	0.5420 (14)	0.4736 (7)	0.101 (4)	0.687 (8)
H28A	0.269746	0.473453	0.468243	0.151*	0.687 (8)
H28B	0.243009	0.639743	0.482490	0.151*	0.687 (8)
H28C	0.183275	0.516023	0.528416	0.151*	0.687 (8)
C24	−0.0276 (6)	0.5901 (14)	0.2767 (7)	0.133 (5)	0.687 (8)
H24A	0.017885	0.604487	0.231128	0.199*	0.687 (8)
H24B	−0.050962	0.489079	0.274766	0.199*	0.687 (8)
H24C	−0.090841	0.653078	0.259808	0.199*	0.687 (8)
C26	0.0834 (11)	0.7670 (11)	0.3672 (11)	0.183 (7)	0.687 (8)
H26A	0.120388	0.763557	0.314619	0.275*	0.687 (8)
H26B	0.026426	0.840144	0.353478	0.275*	0.687 (8)
H26C	0.135088	0.792067	0.426510	0.275*	0.687 (8)
C25	−0.0220 (11)	0.6102 (18)	0.4520 (11)	0.231 (10)	0.687 (8)
H25A	−0.049249	0.511175	0.451164	0.347*	0.687 (8)
H25B	0.026171	0.630006	0.514173	0.347*	0.687 (8)
H25C	−0.082490	0.678083	0.441141	0.347*	0.687 (8)
H3	0.147 (2)	0.399 (3)	0.290 (2)	0.037 (7)*	
H5	0.491 (2)	0.035 (4)	0.571 (2)	0.048 (8)*	
H1	0.625 (3)	0.645 (4)	0.285 (2)	0.050 (8)*	
C28A	−0.0441 (7)	0.5301 (14)	0.3463 (16)	0.086 (5)	0.313 (8)
H28D	−0.076611	0.463036	0.295219	0.129*	0.313 (8)
H28E	−0.068231	0.505978	0.404360	0.129*	0.313 (8)
H28F	−0.065948	0.629095	0.326818	0.129*	0.313 (8)
O5A	0.0714 (8)	0.5189 (11)	0.3649 (7)	0.075 (3)	0.313 (8)
C27A	0.1104 (9)	0.6173 (10)	0.4379 (7)	0.061 (3)	0.313 (8)
C25A	0.0502 (12)	0.7738 (16)	0.414 (2)	0.165 (14)	0.313 (8)
H25D	0.078353	0.841867	0.465413	0.248*	0.313 (8)
H25E	0.063707	0.811400	0.353747	0.248*	0.313 (8)
H25F	−0.027408	0.761711	0.407048	0.248*	0.313 (8)
C24A	0.2273 (17)	0.591 (3)	0.465 (2)	0.133 (15)	0.313 (8)
H24D	0.260701	0.656770	0.516101	0.199*	0.313 (8)
H24E	0.240780	0.490800	0.486222	0.199*	0.313 (8)
H24F	0.258158	0.607410	0.409066	0.199*	0.313 (8)
C26A	0.0671 (15)	0.583 (4)	0.5248 (13)	0.184 (15)	0.313 (8)
H26D	0.095278	0.653228	0.575311	0.275*	0.313 (8)
H26E	−0.011687	0.588288	0.507957	0.275*	0.313 (8)
H26F	0.089540	0.485615	0.547460	0.275*	0.313 (8)

### (1R,2S,5S)-N-[(1S)-1-Cyano-2-[(3S)-2-oxopyrrolidin-3-yl]ethyl]-3-[(2S)-3,3-dimethyl-2-[(2,2-trifluoroacetyl)amino]butanoyl]-6,6-dimethyl-3-azabicyclohexane-2-carboxamide methyl <it>tert</it>-butyl ether monosolvate Atomic displacement parameters (Å^2^)

**Table:**

	*U*^11^	*U*^22^	*U*^33^	*U*^12^	*U*^13^	*U*^23^
F3	0.0568 (8)	0.0476 (9)	0.0466 (7)	−0.0173 (7)	0.0125 (6)	0.0103 (6)
O2	0.0304 (7)	0.0502 (9)	0.0207 (6)	−0.0057 (6)	0.0009 (5)	0.0028 (6)
F2	0.0774 (11)	0.0554 (10)	0.0626 (10)	−0.0134 (8)	0.0344 (9)	−0.0182 (8)
F1	0.0467 (7)	0.0512 (9)	0.0614 (9)	−0.0119 (7)	−0.0154 (7)	0.0109 (7)
N2	0.0268 (7)	0.0292 (9)	0.0209 (7)	−0.0007 (6)	0.0029 (6)	0.0007 (6)
N1	0.0291 (8)	0.0358 (10)	0.0202 (8)	−0.0041 (7)	0.0011 (6)	0.0021 (7)
O3	0.0662 (11)	0.0326 (9)	0.0515 (10)	−0.0062 (8)	0.0268 (8)	−0.0101 (7)
O4	0.0767 (12)	0.0669 (13)	0.0258 (8)	0.0359 (11)	−0.0006 (7)	−0.0044 (8)
N3	0.0334 (8)	0.0299 (9)	0.0268 (8)	−0.0039 (7)	0.0023 (7)	0.0018 (7)
N5	0.0417 (10)	0.0575 (13)	0.0252 (9)	0.0101 (9)	−0.0026 (7)	0.0043 (8)
O1	0.1116 (18)	0.0650 (15)	0.0349 (10)	−0.0384 (13)	−0.0147 (10)	0.0176 (9)
C1	0.0381 (11)	0.0349 (12)	0.0335 (10)	−0.0039 (9)	0.0066 (9)	0.0045 (9)
C8	0.0288 (9)	0.0254 (9)	0.0227 (8)	−0.0029 (7)	0.0013 (7)	−0.0021 (7)
C15	0.0264 (8)	0.0279 (10)	0.0238 (8)	−0.0017 (7)	0.0029 (7)	−0.0008 (7)
C16	0.0278 (9)	0.0318 (11)	0.0278 (9)	−0.0034 (8)	0.0007 (7)	−0.0014 (8)
C2	0.0364 (10)	0.0378 (12)	0.0263 (10)	−0.0043 (9)	0.0032 (8)	0.0045 (9)
C3	0.0266 (9)	0.0353 (11)	0.0214 (8)	−0.0001 (8)	−0.0007 (7)	0.0001 (7)
C23	0.0469 (12)	0.0357 (12)	0.0250 (10)	0.0020 (9)	−0.0030 (8)	−0.0007 (8)
C10	0.0328 (10)	0.0368 (11)	0.0242 (9)	0.0046 (8)	−0.0004 (7)	0.0015 (8)
C14	0.0264 (9)	0.0375 (11)	0.0283 (9)	0.0014 (8)	0.0011 (7)	−0.0005 (8)
C17	0.0364 (10)	0.0347 (12)	0.0325 (10)	−0.0079 (9)	−0.0031 (8)	0.0057 (9)
C11	0.0376 (11)	0.0371 (12)	0.0343 (10)	0.0108 (9)	0.0067 (8)	0.0058 (9)
C9	0.0318 (9)	0.0399 (12)	0.0198 (9)	0.0046 (8)	0.0022 (7)	0.0028 (8)
C20	0.0352 (10)	0.0374 (11)	0.0242 (9)	−0.0033 (9)	0.0005 (7)	0.0047 (8)
C19	0.0366 (10)	0.0381 (12)	0.0308 (10)	−0.0027 (9)	0.0050 (8)	0.0089 (9)
O5	0.0424 (18)	0.070 (2)	0.0494 (17)	0.0150 (17)	−0.0005 (14)	−0.0184 (15)
C4	0.0354 (10)	0.0379 (12)	0.0302 (10)	0.0055 (9)	0.0014 (8)	−0.0028 (9)
C21	0.0481 (12)	0.0608 (16)	0.0262 (10)	0.0103 (11)	0.0046 (9)	0.0014 (10)
C12	0.0617 (14)	0.0274 (12)	0.0499 (13)	0.0038 (10)	0.0144 (11)	0.0003 (10)
C5	0.0483 (12)	0.0401 (13)	0.0380 (12)	0.0101 (10)	−0.0064 (10)	0.0011 (10)
C7	0.0486 (13)	0.0352 (12)	0.0434 (13)	0.0050 (10)	−0.0013 (10)	−0.0083 (10)
C22	0.0502 (13)	0.080 (2)	0.0346 (11)	0.0178 (14)	0.0094 (10)	0.0104 (12)
C13	0.0519 (14)	0.0627 (19)	0.0515 (15)	0.0282 (13)	0.0093 (11)	0.0170 (13)
C6	0.0418 (12)	0.0605 (17)	0.0536 (15)	0.0105 (12)	0.0144 (11)	−0.0055 (13)
C18	0.0457 (15)	0.0526 (17)	0.0647 (17)	−0.0178 (12)	−0.0117 (12)	0.0222 (14)
N4	0.0440 (14)	0.094 (3)	0.132 (3)	−0.0278 (15)	−0.0163 (16)	0.043 (2)
C27	0.045 (2)	0.046 (2)	0.064 (3)	0.0086 (17)	0.022 (2)	−0.0032 (18)
C28	0.143 (8)	0.063 (7)	0.066 (4)	0.032 (5)	−0.040 (4)	−0.036 (4)
C24	0.078 (4)	0.185 (11)	0.111 (6)	0.072 (6)	−0.030 (4)	−0.060 (7)
C26	0.220 (13)	0.068 (6)	0.327 (18)	0.028 (7)	0.198 (14)	0.059 (8)
C25	0.205 (13)	0.29 (2)	0.264 (17)	0.148 (14)	0.192 (14)	0.172 (16)
C28A	0.027 (4)	0.057 (7)	0.161 (16)	0.006 (4)	−0.005 (6)	0.013 (8)
O5A	0.056 (5)	0.083 (6)	0.083 (6)	0.017 (5)	0.011 (4)	−0.026 (5)
C27A	0.091 (8)	0.041 (5)	0.060 (6)	−0.005 (5)	0.036 (6)	−0.005 (4)
C25A	0.061 (8)	0.054 (9)	0.34 (3)	0.016 (7)	−0.039 (14)	−0.090 (15)
C24A	0.100 (13)	0.08 (2)	0.16 (2)	0.034 (12)	−0.077 (14)	−0.074 (16)
C26A	0.095 (12)	0.36 (4)	0.116 (14)	−0.01 (2)	0.065 (11)	−0.06 (2)

### (1R,2S,5S)-N-[(1S)-1-Cyano-2-[(3S)-2-oxopyrrolidin-3-yl]ethyl]-3-[(2S)-3,3-dimethyl-2-[(2,2-trifluoroacetyl)amino]butanoyl]-6,6-dimethyl-3-azabicyclohexane-2-carboxamide methyl <it>tert</it>-butyl ether monosolvate Geometric parameters (Å, º)

**Table:**

F3—C1	1.314 (3)	C10—C9	1.508 (3)
O2—C8	1.243 (2)	C14—C11	1.506 (3)
F2—C1	1.337 (3)	C17—C19	1.531 (3)
F1—C1	1.331 (3)	C17—C18	1.484 (3)
N2—C8	1.331 (2)	C11—C12	1.508 (3)
N2—C15	1.468 (2)	C11—C13	1.520 (3)
N2—C9	1.484 (2)	C20—C19	1.519 (3)
N1—C2	1.327 (3)	C20—C21	1.525 (3)
N1—C3	1.460 (3)	O5—C27	1.456 (5)
O3—C16	1.216 (3)	O5—C28	1.376 (10)
O4—C23	1.229 (3)	C4—C5	1.535 (3)
N3—C16	1.348 (3)	C4—C7	1.528 (3)
N3—C17	1.444 (3)	C4—C6	1.529 (3)
N5—C23	1.326 (3)	C21—C22	1.531 (3)
N5—C22	1.455 (3)	C18—N4	1.135 (4)
O1—C2	1.212 (3)	C27—C24	1.534 (8)
C1—C2	1.531 (3)	C27—C26	1.449 (10)
C8—C3	1.531 (3)	C27—C25	1.448 (9)
C15—C16	1.529 (3)	C28A—O5A	1.440 (12)
C15—C14	1.518 (3)	O5A—C27A	1.394 (13)
C3—C4	1.553 (3)	C27A—C25A	1.641 (16)
C23—C20	1.525 (3)	C27A—C24A	1.47 (2)
C10—C14	1.513 (3)	C27A—C26A	1.513 (17)
C10—C11	1.510 (3)		
			
C8—N2—C15	118.82 (16)	N3—C17—C18	110.0 (2)
C8—N2—C9	128.26 (16)	C18—C17—C19	109.19 (19)
C15—N2—C9	112.80 (15)	C10—C11—C13	115.3 (2)
C2—N1—C3	120.48 (17)	C14—C11—C10	60.22 (14)
C16—N3—C17	120.65 (19)	C14—C11—C12	121.58 (19)
C23—N5—C22	113.62 (19)	C14—C11—C13	114.8 (2)
F3—C1—F2	106.9 (2)	C12—C11—C10	122.23 (19)
F3—C1—F1	108.25 (18)	C12—C11—C13	113.0 (2)
F3—C1—C2	111.28 (17)	N2—C9—C10	104.24 (15)
F2—C1—C2	110.81 (19)	C23—C20—C21	102.78 (18)
F1—C1—F2	106.53 (19)	C19—C20—C23	109.96 (17)
F1—C1—C2	112.8 (2)	C19—C20—C21	116.8 (2)
O2—C8—N2	120.71 (17)	C20—C19—C17	113.22 (17)
O2—C8—C3	121.51 (17)	C28—O5—C27	122.8 (6)
N2—C8—C3	117.78 (16)	C5—C4—C3	113.04 (18)
N2—C15—C16	109.67 (15)	C7—C4—C3	107.91 (17)
N2—C15—C14	104.44 (15)	C7—C4—C5	110.2 (2)
C14—C15—C16	110.58 (16)	C7—C4—C6	109.3 (2)
O3—C16—N3	123.7 (2)	C6—C4—C3	107.3 (2)
O3—C16—C15	122.15 (19)	C6—C4—C5	109.06 (19)
N3—C16—C15	114.15 (18)	C20—C21—C22	103.54 (19)
N1—C2—C1	115.23 (17)	N5—C22—C21	102.51 (19)
O1—C2—N1	126.7 (2)	N4—C18—C17	178.8 (4)
O1—C2—C1	118.1 (2)	O5—C27—C24	104.8 (4)
N1—C3—C8	109.22 (16)	C26—C27—O5	99.8 (6)
N1—C3—C4	113.04 (16)	C26—C27—C24	104.2 (9)
C8—C3—C4	113.02 (17)	C25—C27—O5	113.3 (6)
O4—C23—N5	126.7 (2)	C25—C27—C24	115.0 (9)
O4—C23—C20	124.6 (2)	C25—C27—C26	117.9 (10)
N5—C23—C20	108.67 (19)	C27A—O5A—C28A	105.5 (10)
C11—C10—C14	59.74 (14)	O5A—C27A—C25A	110.3 (10)
C9—C10—C14	108.39 (16)	O5A—C27A—C24A	104.7 (12)
C9—C10—C11	119.22 (18)	O5A—C27A—C26A	110.6 (13)
C10—C14—C15	108.01 (15)	C24A—C27A—C25A	126.8 (14)
C11—C14—C15	118.91 (18)	C24A—C27A—C26A	106.8 (18)
C11—C14—C10	60.04 (14)	C26A—C27A—C25A	96.9 (12)
N3—C17—C19	111.78 (18)		

### (1R,2S,5S)-N-[(1S)-1-Cyano-2-[(3S)-2-oxopyrrolidin-3-yl]ethyl]-3-[(2S)-3,3-dimethyl-2-[(2,2-trifluoroacetyl)amino]butanoyl]-6,6-dimethyl-3-azabicyclohexane-2-carboxamide methyl <it>tert</it>-butyl ether monosolvate Hydrogen-bond geometry (Å, º)

**Table:**

*D*—H···*A*	*D*—H	H···*A*	*D*···*A*	*D*—H···*A*
N1—H1···O4^i^	0.80 (3)	2.06 (3)	2.837 (2)	165 (4)
N3—H3···O5	0.87 (3)	1.99 (3)	2.848 (4)	170 (2)
N3—H3···O5*A*	0.87 (3)	1.95 (3)	2.795 (10)	163 (3)
N5—H5···O2^ii^	0.85 (3)	2.00 (3)	2.845 (2)	176 (3)
C5—H5*C*···O2	0.98	2.51	3.119 (3)	120
C7—H7*C*···O1^iii^	0.98	2.50	3.456 (3)	165
C12—H12*A*···N2	0.98	2.41	3.069 (3)	124
C24—H24*C*···F1^iv^	0.98	2.51	3.418 (11)	154

Symmetry codes: (i) −*x*+1, *y*+1/2, −*z*+1; (ii) −*x*+1, *y*−1/2, −*z*+1; (iii) −*x*+1, *y*−1/2, −*z*; (iv) *x*−1, *y*, *z*.
